# A comprehensive method to develop a checklist to increase safety of intra-hospital transport of critically ill patients

**DOI:** 10.1186/s13054-015-0938-1

**Published:** 2015-05-07

**Authors:** Anja H Brunsveld-Reinders, M Sesmu Arbous, Sander G Kuiper, Evert de Jonge

**Affiliations:** Department of Intensive Care, Leiden University Medical Center, Albinusdreef 2, PO Box 9600, 2300 RC Leiden, the Netherlands

## Abstract

**Introduction:**

Transport of critically ill patients from the Intensive Care Unit (ICU) to other departments for diagnostic or therapeutic procedures is often a necessary part of the critical care process. Transport of critically ill patients is potentially dangerous with up to 70% adverse events occurring. The aim of this study was to develop a checklist to increase safety of intra-hospital transport (IHT) in critically ill patients.

**Method:**

A three-step approach was used to develop an IHT checklist. First, various databases were searched for published IHT guidelines and checklists. Secondly, prospectively collected IHT incidents in the LUMC ICU were analyzed. Thirdly, interviews were held with physicians and nurses over their experiences of IHT incidents. Following this approach a checklist was developed and discussed with experts in the field. Finally, feasibility and usability of the checklist was tested.

**Results:**

Eleven existing guidelines and five checklists were found. Only one checklist covered all three phases: pre-, during- and post-transport. Recommendations and checklist items mostly focused on the pre-transport phase. Documented incidents most frequently related to patient physiology and equipment malfunction and occurred most often during transport. Discussing the incidents with ICU physicians and ICU nurses resulted in important recommendations such as the introduction of a standard checklist and improved communication with the other departments. This approach resulted in a generally applicable checklist, adaptable for local circumstances. Feedback from nurses using the checklist were positive, the fill in time was 4.5 minutes per phase.

**Conclusion:**

A comprehensive way to develop an intra-hospital checklist for safe transport of ICU patients to another department is described. This resulted in a checklist which is a framework to guide physicians and nurses through intra-hospital transports and provides a continuity of care to enhance patient safety. Other hospitals can customize this checklist to their own situation using the methods proposed in this paper.

**Electronic supplementary material:**

The online version of this article (doi:10.1186/s13054-015-0938-1) contains supplementary material, which is available to authorized users.

## Introduction

Critically ill patients are frequently transported between the ICU and other sections of the hospital for diagnostic and/or therapeutic interventions [1-3]. Unfortunately there is an increased risk of an adverse event during intra-hospital transport (IHT) [4]. The first documentation that IHT is potentially dangerous was published in 1970: during transport, arrhythmia occurred in 84% of patients at high risk of cardiovascular events [5]. Subsequent studies reported incidents in 4.2 to 70.0% of critically ill patients during IHT [1-3,6-8]. Incidents were mostly related to equipment failure (39 to 45%) [6-8], physiological deterioration of the patient including hypotension in up to 47% and hypoxia (20 to 29%) [3]. Specific knowledge on the risk of particular incidents during IHT can contribute to improved safety but so far little is known about what kind of incidents occur during intra-hospital transport of critically ill patients.

Measures to reduce incidents include better pre-transport planning, the introduction of standardised procedures related to personnel, organisation and equipment during transport and the use of checklists during the preparation phase [3,6-10]. Indeed, some guidelines on optimal IHT [11,12] are available but they are not easily translated into practical measures to reduce incidents. As an alternative, checklists are practical and can provide tools to improve safety [13]. The aim of our study was to develop a checklist covering the pre-transport preparation phase, the actual transport phase and the ICU reinstallation (post-transport) phase, to improve safety during intra-hospital transport of adult critically ill patients.

## Methods

This study was conducted in a 29-bed, adult patient mixed tertiary ICU at the Leiden University Medical Center (LUMC), the Netherlands. Three complementary methods were sequentially applied to develop the checklist. These consisted of (1) a review of the available literature on IHT guidelines and checklists, (2) an analysis of incidents related to IHT at the LUMC and (3) an inventory of what could go wrong during IHT and how to prevent its accumulation through structured interviews with ICU doctors and ICU nurses. Based upon the study results, a checklist was developed and the feasibility and usability of the checklist were tested during a one-month period.

### Definitions

For the purpose of this study we explicitly divided intra-hospital transport into three phases, and for the literature search we determined whether these three phases were addressed in the guidelines and checklists. Furthermore, we specifically focussed on the separate phases when analysing the reported incidents and in the interviews with doctors and nurses [14].

The pre-transport phase is the phase in which the patient is prepared for transport. The focus is on the patient’s severity of illness and stability, on the kind of monitoring and therapy the patient currently requires and also on what the patient is likely to need during the transport process. The transport phase comprises the transport from the ICU to another department and vice versa as well as the period during the diagnostic or therapeutic procedure. The post-transport phase is the phase when the patient has returned to the ICU, in which ICU monitoring and earlier ICU therapies have to be reinstalled, and the patient has to be stabilised. This phase requires 0.5 to 1 h after transport and must be considered as part of the transport process. An incident is defined as ‘any event or outcome which could have reduced, or did reduce the safety margin for the patient. It may or may not have been preventable and may or may not have involved an error on the part of the health care team’ [15].

#### Review of the literature

Our review of the literature focused on guidelines and checklists on intra-hospital transport of critically ill patients. We searched in PubMed, Embase, Web of Science, COCHRANE, CINAHL, Academic Search Premier and ScienceDirect; from inception until 12 January 2014. The databases were searched for medical literature with the following terms: ‘intensive care’, ‘critical care’, ‘critically ill’, ‘intra-hospital transport’, ‘in-hospital transport’, ‘radiology department’, ‘guideline’ and ‘checklist’. Reference lists of review articles and eligible primary studies were checked to identify cited articles not captured by electronic searches.

### Study selection

Two authors (AB and SK) scrutinised titles and abstracts of all references for possible inclusion. Inclusion criteria were: transport of adult ICU patients in the hospital, checklist and/or recommendations for IHT. Excluded were articles related to paediatric critical care, inter-hospital transport, reviews and editorials. Full text articles were examined and any disagreement was resolved by a third author (SA).

### Data abstraction

The following data were abstracted from the studies with guidelines or checklists: author/research group, year of publication, country and recommendations and checklist items related to the pre-transport-, transport- and post-transport phase.

#### Analysis of incidents related to transport

We collected and analysed IHT incidents in our hospital to learn about the types and contributing factors of IHT incidents. In our ICU all incidents are submitted to an electronic incident reporting system. All routinely registered transport-related incidents were analysed and categorised with respect to type, phase of occurrence and contributing factors in the period from 2006 to 2009. Subsequently, over a 12-month period in 2012 we specifically asked ICU physicians and ICU nurses to report all incidents occurring during intra-hospital transport. A questionnaire was developed to collect these incidents. Incidents were predefined and categorised as airway, breathing, circulation, disability, exposure and other. Also, a free-text field allowed the reporter to give a description of the situation during transport, perceived causes and actions that were taken. Incidents were analysed with respect to type, circumstances and contributing factors.

#### Interviews with experts in the field of intensive care

Structured interviews based on findings from the literature and collected incidents were undertaken with ten ICU physicians and fifteen ICU nurses. The interviews followed a questionnaire containing 53 questions on what could go wrong during the three phases of IHT and how to prevent it. Questions were related to equipment, patient physiology, monitoring, medication and fluid management; and covered all three transport phases. Additionally, for the transport phase questions focused on logistics and communication with the other department, and registration of vital signs. For the post-transport phase the focus was on the reinstallation of ICU therapies and monitoring and on the stabilization of the patient. A detailed overview of the questions used for the structured interview can be found in Additional file 1.

#### Development of the checklist

The information gathered from the review of the literature, the analysis of transport-related incidents and the interviews with experts in the field were combined to develop the checklist. Checklist items were structured according to the different phases of transport. The checklist was introduced to ICU physicians and ICU nurses and was implemented in the Patient Data Management System of our ICU to be used in daily practice.

#### Feasibility and usability

The checklist was used by the ICU for one month, whereupon we collected data to investigate the feasibility and usability of the checklist. Nurses were asked to fill in a questionnaire after each transport documenting their experiences using this checklist. The following data were collected: overall rating of the checklist, the time it took to fill in the checklist, relevance of the questions, logistics of the filling in of the checklist, and questions that were felt to be lacking. The questionnaire is listed in Additional file 2.

### Ethical approval

The Medical Ethics Committee of the LUMC waived the need for ethical evaluation of the study due to the observational nature of the study. Consequently, the need for informed consent was not applicable.

## Results

### Review of the literature

In total eleven guidelines [11,12,16-24] and five checklists on IHT [25-29] were identified in the literature. The guidelines were developed in USA, Europe, India, Australia and New Zealand and described recommendations for intra-hospital transport as well as for inter-hospital transport. In the guidelines some basic principles regarding transport were defined for example, that a hospital transport protocol should be present [11,16-18,21,22,24] and that the patient should receive the same level of basis physiologic monitoring during IHT as they received in the ICU [12,17-19,23]. Three phases of transport were recognized. For each phase recommendations could be subdivided into categories namely (i) use of (monitoring) equipment, (ii) patient physiology, (iii) medication and fluids, (iv) organization and planning. The pre-transport phase was most extensively described. In this phase, recommendations were related to the use of a transport trolley, equipment to secure an airway, and preparation of monitoring, medication and fluids. With respect to patient physiology, a careful evaluation of the risk-benefit ratio should be made by the physician [11,16-24] and special attention should be paid to the indication for transport [11,12,17,18,23,24]. Other recommendations included planning of personnel with the suggestion that a minimum of two qualified staff members, an ICU nurse and ICU physician, should accompany the patient [11,12,16-24] and the need for clear communication to ensure that the patient is expected at the destination department [16,20,22-24] and to confirm that the receiving party is ready [11,12,20,23,24].

In the transport phase an important goal should be to continue monitoring during the transport as well as during the diagnostic or therapeutic procedure [11,17,18] and to check and record the patient’s vital signs on a regular basis, at least every 15 minutes [16,24]. Furthermore, medication and fluid management and maintenance of physiologic stability should be of key importance.

Back in the ICU, after installation and stabilization of the patient, it is essential to check monitoring and medication and to document the course of the transport in the medical chart. With respect to the latter, attention should be paid to the status of the patient during and after transport [11,12,16-18,23,24] and also to the events and interventions that occurred during transport [12,16-18,20,24]. All the transport equipment should be cleaned and plugged back in the main power supply to ensure that the equipment is available for another transport to the receiving department for a diagnostic or therapeutic intervention.

In the literature, five checklists for intra-hospital transport of critically ill patients were found [25-29], of which one was specifically developed for obese patients [29]. The main focus of the checklists was on the pre-transport phase. Only the checklist developed by Jarden [27] also described items for the transport and post-transport phase. Checklist items in the pre-transport phase related to the patient, monitoring equipment, communication and quality of the team. Before transport, the clinical stability of the patient [26-28] and the necessity of the transport should be assessed [28]. Medication, fluids and the equipment should be checked including transport trolley, monitoring devices, and additional equipment [25-29]. Items related to planning and organization should also receive attention [26,28,29]. For example, in order to guarantee a safe transport, items were formulated with respect to the composition of the transport team, namely the presence of a physician [27] and a minimum number of ICU nurses [26].

During transport, when the patient has arrived at the destination department, various items should be checked and ensured. First, the continuity of the oxygen supply and the electronic supply for transport trolley and medication pumps should be checked [27]. Furthermore, vital signs and administration of medication should be registered frequently.

Upon return in the ICU, it is essential to reinstall respiratory support devices, medication and monitoring, and to describe in the medical chart the complications that have occurred during transport and to recheck the used equipment [27]. An overview of the content of the published checklists is shown in Table 1.

**Table 1:** **An overview of the content of published intra-hospital (IHT) checklists**

**Author**	**Pope [** 28 **]**	**Fanara [** 26 **]**	**Jarden [** 27 **]**	**Roland [** 29 **]**	**Choi [** 25 **]**	**Current checklist** ^**a**^
Year of publication	2003	2010	2010	2010	2011	LUMC
**Pre-transport**						
Necessity of transport is confirmed	+					
Patient assessment pre-transport		+	+			
Wrist band patient or consent form		+	+	+	+	
Transport team is notified	+	+	+			
Equipment and materials are gathered	+	+	+	+	+	+
Check sufficient oxygen level		+		+		+
Extra intravenous fluid and medication	+	+	+	+		+
Check sufficient intravenous medication	+	+	+	+		+
Stop enteral feeding and enteral insulin						+
Check tubes and lines		+	+	+	+	+
Check and set monitor alarms		+	+			+
Check and set transport ventilator alarms		+				+
Insert intravenous cannula in case of computed tomography with contrast						+
Preparation and equipment adapted to procedure (magnetic resonance imaging)		+				+
Fill in magnetic resonance imaging safety questionnaire						+
Register baseline vital signs		+/−	+			+
Receiving department is notified	+					+
Transport route is clear		+				
**During transport**						
Check and plug in equipment at destination			+			+
Registration of administered fluids/medication			+			+
Registration vital signs every 20 minutes			+			+
**Post-transport**						
Start enteral feeding and enteral insulin						+
Turn on humidifier						+
Change HME filter						+
Change suction bag if used			+			+
Complement transport bag						+
Report occurred incidents/events			+			+
Re-check equipment and materials			+			+

^a^Current checklist Leiden University Medical Center (LUMC) refers to the final checklist that was based on reviewing the available literature on IHT checklists and guidelines, an analysis of transport related incidents and a structured interview with ICU physicians and ICU nurses. HME, heat and moisture exchanger.

### Analysis of incidents related to IHT

Over a 36-month period, a total of 5,937 incidents were reported in our incident registration system, of which 118 incidents (2.0%) were IHT related. Of the 118 IHT incidents 38% occurred in the pre-transport phase, 47% in the transport phase and 15% in the post-transport phase. In the pre-transport phase most reported incidents were related to equipment and organizational issues. Examples of equipment-related incidents were: low battery of the ventilator and/or medication pumps, use of a mechanical ventilator not suitable for the MRI and an empty oxygen tank. Examples of organization-related incidents were inappropriate preparation of the patient leading to delay of transport or inadequate communication with the receiving department.

Also in the transport phase most reported incidents were related to equipment and organisation. Examples of equipment incidents during this phase included failure of the transport trolley and its monitor. Examples of the organisational incidents were in availability of CT or MRI equipment. Post-transport, most reported incidents were related to airway and respiratory management, such as failure to install adequate oxygen level or to reconnect the humidifier of the ventilator. An overview of the most common incidents is shown in Table 2.

**Table 2:** **Top ten most commonly reported intra-hospital transport (IHT)-related incidents**

**Top 10 routinely registered IHT related incidents** ^**a**^	**Pre-transport**	**During transport**	**Post-transport**	**Total**
Equipment malfunction	9	24	1	34
Preparation before transport	30	0	0	30
Lack of communication with radiology department	1	5	0	6
Dislocation of intravenous lines and tubing	0	12	1	13
Oxygen tank empty	4	4	0	8
Increase need vasopressor or inotropics	0	3	0	3
Equipment not available at radiology department	0	5	0	5
Lack of documentation in medical chart	0	0	2	2
Failure reconnect humidifier on ventilator	0	0	11	11
Hypoglycemia	0	0	1	1
**Top ten prospectively collected IHT-related incidents** ^**b**^				
Equipment malfunction	7	24	2	33
Preparation before transport	6	5	0	11
Lack of communication with radiology department	5	5	0	10
Dislocation intravenous line	0	7	2	9
Oxygen tank empty	4	2	0	6
Increase need vasopressor or inotropics	5	15	6	26
Low blood pressure^§^	21	44	18	83
Hypoxia^§^/increased oxygen demand	5	18	12	35
Increased need sedatives or opiods due to agitation	2	17	2	21
Hypertension^§^	2	9	3	14

^a^Analysis of transport-related incidents that were identified from routinely collected incidents in an elecronic incident reporting system in Leiden University Medical Center. ^b^For 12 months all incidents occurring during intra-hospital transport were prospectively collected. ^§^No definitions were used to define hypotension, hypertension and hypoxia. Physicians and nurses were able to judge whether it deviated.

In 2012, we prospectively collected transport-related incidents. In this period, 503 transports to the radiology department were undertaken. In 334/503 (66%) of IHTs an ICU physician and ICU nurse accompanied the patients to the radiology department. In 133/503 (27%) of IHTs three ICU staff members, an ICU physician and two ICU nurses and in 16/503 (3%) four ICU staff members, two ICU physicians and two ICU nurses accompanied the patient. When the patient was not intubated the nurses sometimes accomplished the transport without a physician 20/503 (4%). The median duration of the transport was 55 minutes (range 10 to 305 minutes). In 77% the reason for the IHT was to perform computed tomography (CT) and in 10% angiography.

In 133 of the 503 transports (26%), one or more incidents occurred, and in total, 358 incidents were reported. Incidents occurred in the transport phase (215/358, 60%), in the pre-transport phase (80/358, 22%) and in the post-transport phase (63/358, 18%). The ten most frequently reported incidents during transport are shown in Table 2. In the transport phase the incidents were related to hemodynamic instability, respiratory instability, equipment dysfunction and increased need of medication. In the pre-transport and post-transport phase incidents were related to hemodynamic instability. The lack of communication with the radiology department before and during transport also occurred regularly.

### Interviews with experts in the field of intensive care

Ten physicians and fifteen nurses were interviewed to discuss the findings from the literature and the collected incidents. A transport protocol existed in our hospital but 90% of the physicians and 73% of the nurses were not familiar with the protocol. The protocol described the composition of the accompanying team, the monitoring and respiratory equipment to be used, and the medication and additional equipment that should be available during transport.

Incidents considered most important by physicians and nurses in the pre-transport phase were an empty oxygen tank, lack of sufficient intravenous access, missing equipment, trolley failure, inadequate length of intravenous tubing and miscommunication with the radiology department. In the transport phase, nurses and physicians mentioned potential incidents such as dislocation of an intravenous cannula or endotracheal tube, low battery in the pumps, impaired view of the patient in the radiology department and patient instability. In the post-transport phase patient instability and incorrect reinstallation of respiratory support and medication were commonly reported.

To enhance a safer transport, several improvement measures were suggested by physicians and nurses, such as introduction of a checklist for the three phases of transport and standardisation of the transport procedure and improved communication with the radiology department. A list of recommendations can be found in Table 3. Furthermore, the physicians and nurses indicated that they would feel more confident if they received more education and practical training.

**Table 3:** **Recommendations from ICU physicians and ICU nurses**

	**Recommendations**
**Team**	Ventilated patient at least one ICU physician and one ICU nurse
	Not ventilated patient and:
	o ≤ 1 inotropic, one ICU nurse
	o ≤ 1 inotropic, respiratory insufficient and arrhythmia, one ICU physician and one ICU nurse
**Education**	Focus on how to operate equipment of transport trolley
	More education for ICU physicians and ICU nurses to execute transport of ICU patients
**Equipment and materials**	Equipment on trolley is equal to equipment in the ICU
	Check equipment and materials prior to transport
	Check extra length of intravenous lines for magnetic resonance imaging prior to transport
	Check and calculate oxygen level in oxygen tank
	Defibrillator is standard equipment on transport trolley
	Check all equipment on transport trolley
	Batteries are fully charged prior to transport
**Organization and procedure**	Introduction of an intra-hospital checklist
	Formal training in transport procedure to MRI
	Standard Operating Procedure
	Standardization of IHT procedure
**Communication**	Confirm appointment with the other department prior to transport
	Improve communication with the other department to prevent incidents during transport
	Debriefing with ICU physician and ICU nurse after transport
**Medication**	Check and prepare intravenous medication prior to transport
	Extra intravenous medication and intravenous fluids

Recommendations suggested by ICU physicians and ICU nurses when they were interviewed to discuss safety and hazards of IHT and the findings from the literature and the collected incidents.

### Development of the checklist

Based on the literature, we chose the checklist of Jarden [27] as a base to develop our own checklist. The other four checklists were used to complement our new checklist. All the checklists had several items in common such as check equipment/materials [25-29], medication [26-28] and intravenous access [25-29]. We included these items in our checklist. One item, only found in the checklist by Pope was ‘whether the receiving department is notified’ and we included this item also in our checklist [28]. An overview of the items of the published checklists is shown in Table 1.

**Figure 1:**
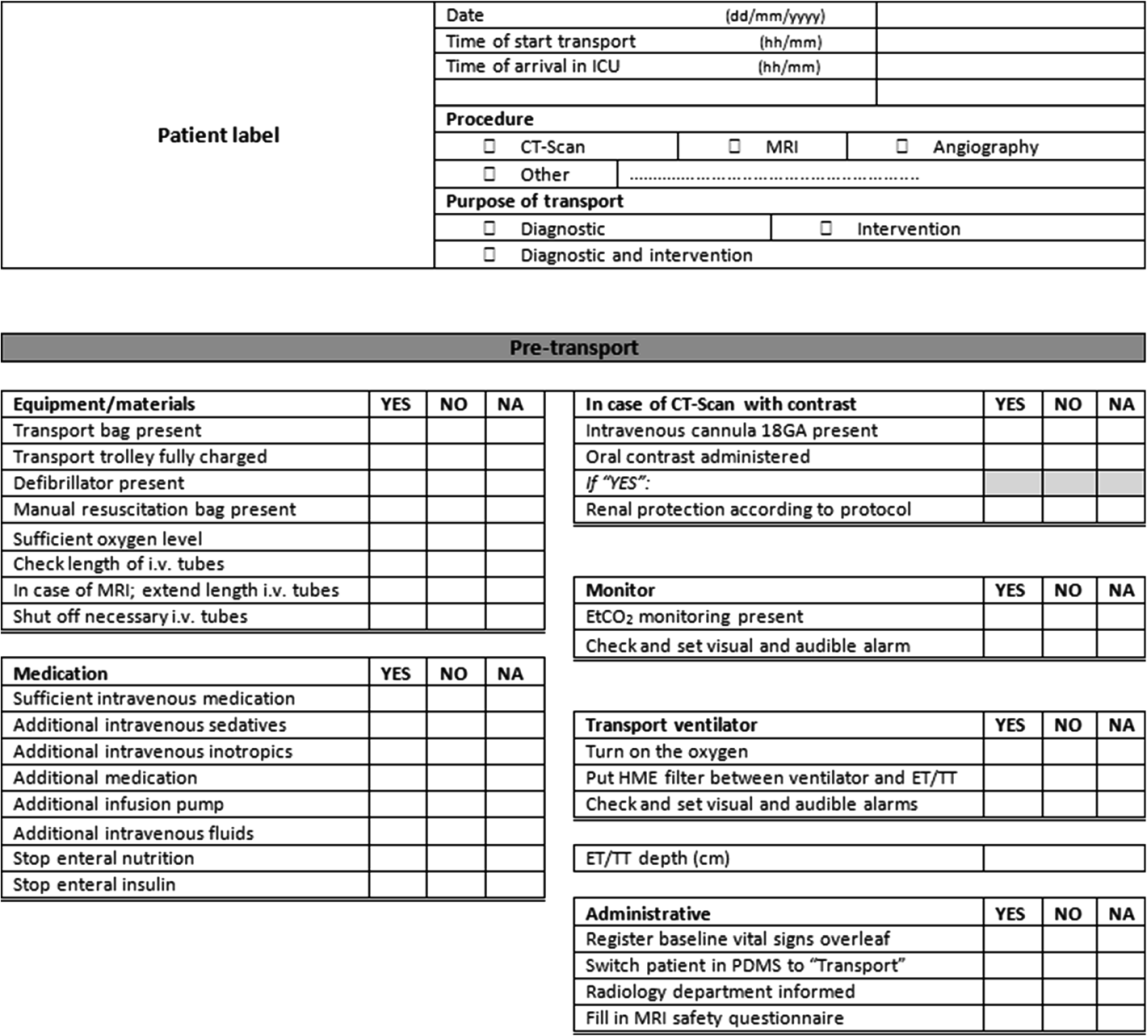
Newly developed Leiden University Medical Center (LUMC), checklist side one. i.v., intravenous; MRI, magnetic resonance imaging; EtCO_2_, end tidal CO_2_; HME, heat and moisture exchanger; ET/TT, endotracheal tube/tracheal tube; PDMS, Patient Data Management System.

**Figure 2:**
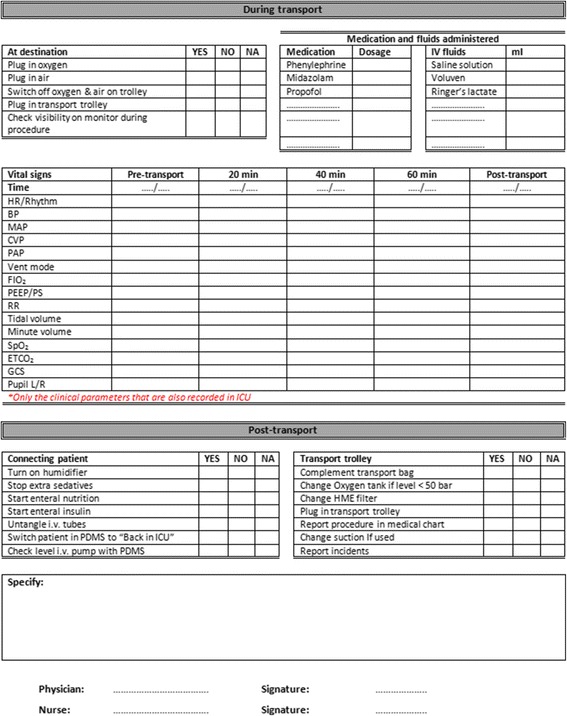
Newly developed Leiden University Medical Center (LUMC) checklist, side two. i.v., intravenous; HR, heartrate; BP, bloodpressure; MAP, mean aterial pressure; CVP, central venous pressure; PAP, pulmonary artery pressure; Vent mode, ventilation mode; FIO_2_, fraction of inspired oxygen; PEEP/PS, postive end-expiratory pressure/pressure support; RR, respiratory rate; SpO_2_, peripheral capillary oxygen saturation; EtCO_2_, end tidal CO_2_; GCS, Glasgow coma scale; PDMS, Patient Data Management System; HME, heat and moisture exchanger.

The final checklist developed as described above is presented in Figures 1 and 2. The basic principle of this checklist was to guide the physician and nurses through the different phases. In the pre-transport phase the focus is on required equipment, preparation of extra medication and intravenous fluids and checking of procedures such as the use of contrast fluid and kidney protection. In the transport phase the focus is on the destination department with attention for the following items: plugging in the oxygen, monitoring equipment and keeping sight of the monitor during the procedure and registration of vital signs, and medication and intravenous fluids. In the post-transport phase it is important to connect the patient to the equipment in the ICU with specific attention to switching on the humidifier, nutrition, insulin and checking the correct dose of medication via the perfusor. Also, to assure that required equipment is ready for use on the next trip, the transport trolley and transport bag should be checked and connected to the power supply. Finally, documentation in medical charts including registration of incidents should be checked.

### Feasibility and usability

In order to investigate the feasibility and usability of the checklist, data were collected over a one-month period using the checklist. During this month, 41 transports were made to the radiology department. In 29 of these transports, the checklist was used and a questionnaire was later filled in by the nurses about their experiences using the checklist. Reasons for not using the checklist during transport were either due to forgetfulness of the team to use it (5/29) or to the urgency of the transport (7/29). The time it took to fill in the checklist was on average 4.5 minutes per phase (range 3 to 10). Nurses stated that the user friendliness of the checklist was good, it was comprehensive and complete, it reduced the chance of forgetting things, and it was easy to apply because it was implemented in the Patient Data Management System. A point of criticism was the documentation of vital signs every 20 minutes on the paper-based checklist that was used in the transport phase. This was considered time consuming. Digitally input documentation was preferred. Items that were missed in the checklist were information on the completeness of the transport bag and patient assessment in the pre- and post-transport phase. Information on the transport phase and post-transport phase was filled in after the transport.

## Discussion

We developed a checklist to improve safety of intra-hospital transport by using three complementary methods: a review of the available guidelines and checklists in the literature, an analysis of transport-related incidents and an inventory of what could go wrong during IHT and how to prevent it by interviews with ICU doctors and nurses. Importantly our checklist includes three phases of intra-hospital transport. Furthermore, we propose that our methods of local modification of an existing checklist on IHT may be a useful procedure for any hospital aiming at improving safety of intra-hospital transport.

The basic principles for intra-hospital and inter-hospital transport are the same, namely to ensure safety during this potentially dangerous transport [18]. We were specifically interested in intra-hospital transports because they occur frequently on the ICU and because the number of incidents during these transports is still very high. Our checklist is based on an earlier checklist by Jarden [27]. This is the only checklist that discerns three different transport phases. In other checklists the focus was only on the pre-transport phase namely to check the patient and equipment before transport. If the patient is checked before transport it lowers the risks of incidents during transport. However, patient transport is not limited to the pre-transport phase. It is essential that the entire transport process of critically ill patients is covered from start to end.

We wanted to adapt the checklist of Jarden [27] to our own situation. It is often necessary to customise a checklist because aspects of the checklist may not be suitable to a specific local situation. Also in our case, some of our hospital policies and procedures differed from the described checklist items. Therefore, ICUs need to customize the available checklists to their own situation taking into account the hospital procedures and circumstances in which a transport will be conducted.

A comprehensive method was used to develop the checklist. This included a review of the literature for available guidelines and checklists, an analysis of incidents related to transport in our hospital and an inventory of ICU physicians’ and nurses’ expert opinion over IHT. Due to this approach, we obtained different types of knowledge available on the subject and we were better able to build a comprehensive and practical checklist. This approach is supported by Hales *et al*. [30] who stated that peer-reviewed guidelines and evidence-based best practice should be considered to form the body of a checklist and that checklists should also reflect the local hospital and institution policies and procedures.

There are some differences between the Jarden checklist [27] and ours. We added some items that are specifically related to our local situation and some that are a more generic addition for checklists on IHT. For example, in the pre-transport phase checking the availability of sufficient intravenous medication was added. While Jarden’s checklist included a patient assessment and documentation section in the pre-transport phase, we eliminated many of these items because this information can be found in our Patient Data Management System. We added a few items to the checklist that were specific for our IHT policy. Examples of these are extending the length of intravenous tubing, hyper hydration for kidney protection and an MRI safety questionnaire for transport to MRI. In the post-transport phase the focus was on connecting the patient to the available equipment in the ICU and on checking the rate of administration of intravenous pumps with the Patient Data Management System. These items were important for our ICU due to frequently reported incidents that decreased patient safety.

General guidelines and checklists provide guidance in developing a local checklist. The concept of local adaptation of the transport checklist developed by Jarden [27] was not previously described. In our opinion, customising a checklist according to local policies and procedures improves the commitment of nurses and physicians to use this checklist.

A checklist can be seen as an important instrument to avoid incidents. It is of added value if it is introduced accompanied with education and training. Barriers to using checklists in healthcare are related to operational and cultural aspects [13]. Filling in a checklist adds to the nurse’s workload. However, in our small feasibility study, it only took 4.5 minutes (range 3 to 10) per phase and it appeared that nurses were on the whole positive about using a transport checklist.

Our study has a few limitations. First, we have not yet investigated whether our checklist indeed decreases the number of IHT-related incidents and improves safety. This will be the subject of future research. Furthermore, the checklist is by definition most useful in our specific hospital because it is customized to the local hospital and ICU procedures and protocols. Third, while we implemented the pre- and post-transport phase checklist into the Patient Data Management System, the checklist items in the transport phase are still registered on paper (vital signs, medication and fluids). This may result in a potentially lower adherence during this phase.

A strong point of our study was the comprehensive way we developed the checklist. Particularly our inventory of what could go wrong during IHT and how to prevent it, which we achieved through interviews with ICU doctors and nurses, will have contributed to a clinically relevant checklist and to the applicability and acceptance of the checklist in daily practice by ICU doctors and nurses. We think that this checklist can contribute to the safety of ICU patients that need to be transported during their ICU stay. However, to confirm this, the next step to be taken is testing and evaluating the efficacy of the checklist: is patient safety increased with the checklist and are ICU nurses and ICU physicians satisfied using it in daily practice? Our checklist, though specifically adapted for one hospital, can be used in other hospitals as well. Each hospital should assess whether the items from the checklist are applicable to their specific situation. If necessary, local modifications can be made.

## Conclusion

In conclusion, we applied a comprehensive approach to develop an intra-hospital checklist for safe transport of ICU patients to another department and back to the ICU. This checklist is not only based on available guidelines and checklists in the literature but also on reported incidents and expert opinions of ICU physicians and nurses. This resulted in a checklist that is a framework to guide ICU physicians and nurses through intra-hospital transport and provides continuity of care to enhance patient safety.

## Key messages

- A comprehensive method was applied to develop a checklist which can be used to increase the safety of intra-hospital transport of critically ill patients.
- The checklist covers the transport of critically ill patients from the start until the end of the process, including all three transport phases.
- Customizing the checklist according to local policies and procedures - using the comprehensive method suggested in this study - is important to improve the commitment of nurses and physicians.

## Additional files

Additional file 1:
**Questionnaire used for structured interview of ICU physicians and ICU nurses.**
Additional file 2:
**Questionnaire used to assess feasibility and usability of current checklist Leiden University Medical Center (LUMC).**

## Abbreviations

CT: computed tomography
IHT: intra-hospital transport
LUMC: Leiden University Medical Center
MRI: magnetic resonance imaging
