# Supplementary material for: A comprehensive method to develop a checklist to increase safety of intra-hospital transport of critically ill patients
Source: Crit Care. 2015 May 7;19(1):214. doi: 10.1186/s13054-015-0938-1 (PMC4438434; doi:10.1186/s13054-015-0938-1)
Supplement: Additional file 1: — Questionnaire used for structured interview of ICU physicians and ICU nurses. [file 13054_2015_938_MOESM1_ESM.docx]

Appendix 1: Questionnaire used for the structured interview of ICU physicians and ICU nurses for intra-hospital transport (translation from Dutch version)

**Part A. Introduction and general questions**

1. Profession

- ICU Nurse
- Student ICU Nurse
- Resident
- Fellow
- ICU physician

1. Employment at ICU

- < 1 year
- 1 to 5 year
- 5 to 10 year
- > 10 year

1. How often do you transport patients to the radiology department per month?
2. Is there a protocol to transport patients?

- Yes
- No

If ‘Yes’, do you know the contents of the protocol?

- Yes
- No
- NA

Do you examine the protocol before transport?

- Never
- Sometimes
- Often
- Always

If you examine the protocol, what is the reason for examining the protocol?

1. How would you grade ‘I am afraid to transport patient to the radiology department’ on a scale from 0 “very scared” to 10 “not scared”.
2. How would you grade ‘I feel confident to transport the patient to the radiology department’ on a scale from 0 “totally not confident” to 10 “very confident”.
3. What is the most important factor as to why you feel afraid or not confident during transport?

**Part B. Questions related to the pre-transport phase**

1. Before transport is the destination clear?

- Yes
- No

If ‘Yes’, do you know the best route through the hospital?

- Yes
- No

If ‘No’, please specify …………………………..

1. How often is the room number at destination incorrect?
2. How many ICU staff members are sufficient to accompany the patient to the radiology department if:

The patient is not intubated, please specify …………………………..

The patient is intubated, please specify …………………………..

1. The following questions refer to which and how many ICU staff members, in your opinion, should accompany the patient during transport in the following situations.
2. The patient is not intubated and who should accompany (nurse, resident, fellow or intensivist):

- Number of inotropic drugs < 1
- Number of inotropic drugs > 1
- Agitated patient
- Respiratory failure
- Recent arrhythmia

1. The patient is intubated and who should accompany (nurse, resident, fellow or intensivist):

- Number of inotropic drugs < 1
- Number of inotropic drugs > 1
- Agitated patient
- Recent arrhythmia
- Haemodynamic failure
- External equipment (e.g. ECMO or IABP)

1. Several devices are used during transport. Which of these devices are difficult to use?

- Transport monitor
- Transport ventilator
- Suction device
- Air tank
- Oxygen tank

1. How would you grade ‘the operating controls’ of the following devices ‘easy to use’, ‘difficult’ or ‘neutral’?

- Transport monitor
- Transport ventilator
- Suction device
- Air tank
- Oxygen tank

1. Which problems are you dealing with in operating the devices?
2. What would you, at minimum, monitor during transport?

- Monitor ECG
- Invasive blood pressure
- Respiratory rate
- Pulse oximetry
- Non-invasive blood pressure
- Central venous pressure
- Intra cranial blood pressure
- Pressure arterial pulmonary
- Electroencephalogram

1. Do you check the level of oxygen in the oxygen tank before transport?

- Never
- Sometimes
- Often
- Always

1. What is in your opinion the minimum level of oxygen in the oxygen tank before transport?
2. Do you take the transport bag with you during transport?

- Yes
- No

1. Did you ever use the transport bag (equipment and drugs for emergency use)?

- Yes
- No

1. Did you ever miss something in the transport bag?

- Yes
- No

If ‘Yes’, please specify …………………………………

1. Do you always take the defibrillator with you?

- Yes
- No

If ‘Yes’, please specify …………………………………

If ‘No’, please specify …………………………………

1. Do you check if there is a sufficient amount of drugs available during transport?

- Never
- Sometimes
- Often
- Always

1. Do you take extra intravenous medication during transport?

- Yes
- No

If ‘Yes’, please specify which medication:

- Inotropic
- Analgesia
- Sedative
- Muscle relaxant
- Not applicable
- Other, namely …………………………………

1. When extra intravenous medication was taken, have you ever experienced that you don’t have enough medication?
2. How would you grade how often ‘there is insufficient medication’ on a scale from 0 “never” to 10 “every transport”?
3. Do you take pre-prepared medication?

If ‘Yes’ please specify which medication …………………………………

1. Do you take extra intravenous fluids?

In case of ‘Yes’ please specify which fluids and how many …………………………………

1. In case of CT-Scan or MRI do you check the length of the intravenous tubes?

- Never
- Sometimes
- Often
- Always

1. Before transport, discussion takes place between physician and nurse regarding the situation of the patient?

- Never
- Sometimes
- Often
- Always
- Not applicable

1. Who is notified that you are “on transport”?

- Colleague
- Head unit
- None
- Other, namely …………………………………

1. Do you inform the radiology department?

- Never
- Sometimes
- Often
- Always

1. Which incidents occur frequently in the pre-transport phase?
2. Which improvement measures should be taken in this phase?

**Part C. During transport questions**

1. Do you record the following data during transport?

- Vital signs
- Given medication
- Given intravenous fluids

If more than 1 ‘Yes’, how do you record this?

1. Do you discuss who is responsible to check the monitor in case of:
2. During transport?

- Yes
- No

1. During the procedure in the radiology department?

- Yes
- No

1. How is the visual on the transport monitor;
2. During transport at from the ICU to the radiology department and vice versa

- None
- Insufficient
- Sufficient
- Good
- Excellent

1. During the procedure in the radiology department

- None
- Insufficient
- Sufficient
- Good
- Excellent

1. Do you give extra sedation to the patient during transport?

- Never
- Sometimes
- Often
- Always

Please specify the reason, …………………………………

1. Do you give extra analgesia during transport?

- Never
- Sometimes
- Often
- Always

Please specify the reason, …………………………………

1. Do you give extra muscle relaxant to the patient during transport?

- Never
- Sometimes
- Often
- Always

Please specify the reason, …………………………………

1. The following questions refer to the cooperation with the radiology department.
2. Is the radiology department ready when you arrive?

- Never
- Sometimes
- Often
- Always

1. Personnel of the radiology department are helpful during the procedure?

- Never
- Sometimes
- Often
- Always

1. Can you always plug in the oxygen and air?

- Never
- Sometimes
- Often
- Always

1. How would you grade the technical skills of the personnel on a scale from 0 (poor) to 10 (excellent)?
2. How would you grade the communication skills of the personnel on a scale from 0 (poor) to 10 (excellent)?
3. Which incidents occur frequently in the per-transport phase?
4. Which improvement measures should be taken in this phase?

**Part D. Post-transport questions**

1. In case of transport of the patient, do you report this in the medical chart?

- Yes
- No

If ‘Yes’, what do you report (more answers possible)

- Indication of transport
- Duration of the transport
- Patient status during transport
- Action take in case of haemodynamic or respiratory failure
- Incidents and complications
- Extra intravenous medication given

If ‘No’, please specify the reason …………………………………

1. Which incidents occur frequently in the post-transport phase?
2. Which improvement measures should be taken in this phase?

**Part E. General questions**

1. Which transport related problems did you sometimes discover after transport of the patient?
2. In which circumstances would you refuse to transport the patient?
3. Would you refuse to transport the patient in the following situations?
4. Positive End Expiratory Pressure

- Yes
- No

If ‘Yes’, please specify at what level?

1. Oxygen

- Yes
- No

If ‘Yes’, please specify at what level?

1. Inotropic

- Yes
- No

If ‘Yes’, please specify at what level?

1. Do you think there is enough knowledge to ensure the safety during transport of patients if the transporter is an:
2. ICU Physician

- Yes
- No

1. ICU nurse

- Yes
- No

If ‘No’, which improvement measures should be taken?

1. a. An examination is required before an ICU physician can accompany the patient?

- Yes
- No

1. An examination is required before an ICU nurse can accompany the patient?

- Yes
- No

1. How would you grade your expertise in transport of critically ill patients on a scale from 0 (poor) to 10 (excellent)?
2. In your opinion which personnel (ICU nurse, ICU physician or both) is responsible for the following tasks;
3. Preparation of the patient
4. Coordination of the transport
5. Completion of the transport bag
6. Rechecking equipment and completion of the transport trolley
7. Several solutions can be introduced to enhance a safer transport. In your opinion, do the following solutions contribute to a safer transport?

- Extending protocol
- Checklist
- More education
- Transport nurse
- Other, namely …………………………………

Legend Appendix I
