# Supplementary material for: A comprehensive method to develop a checklist to increase safety of intra-hospital transport of critically ill patients
Source: Crit Care. 2015 May 7;19(1):214. doi: 10.1186/s13054-015-0938-1 (PMC4438434; doi:10.1186/s13054-015-0938-1)
Supplement: Additional file 2: — Questionnaire used to assess feasibility and usability of current checklist Leiden University Medical Center (LUMC). [file 13054_2015_938_MOESM2_ESM.docx]

Appendix 2: Questionnaire used to assess feasibility and usability of current checklist LUMC (translation from Dutch version)

**Part A. Content of the Checklist**

1. Did you miss questions in the checklist?
2. In the pre-transport checklist?

- Yes
- No

If ‘Yes’, please specify …………………………..

1. In the transport checklist?

- Yes
- No

If ‘Yes’, please specify …………………………..

1. In the post-transport checklist?

- Yes
- No

If ‘Yes’, please specify …………………………..

1. Does the checklist contain unnecessary questions?

- Yes
- No

If ‘Yes’, please specify …………………………..

1. Did you skip checklist items while you used the checklist?

- Yes
- No

If ‘Yes’, please specify …………………………..

1. What is the reason for skipping these checklist items?

**Part B. User friendliness**

1. Was it easy to fill in the checklist?

- Yes
- No

1. Can you describe in your own words what you find useful or impractical for filling in the checklist?
2. When did you fill in the transport checklist?
3. The pre-transport checklist?

- Before transport
- During transport
- After transport

1. The transport checklist?

- Before transport
- During transport
- After transport

1. The post-transport checklist?

- Before transport
- During transport
- After transport

1. Did you have sufficient time to fill in the checklist?
2. The pre-transport checklist?

- Yes
- No

1. The transport checklist?

- Yes
- No

1. The post-transport checklist?

- Yes
- No

1. If ‘No’ for question 8, please specify …………………………..
2. What is your estimation of time you needed to fill in the checklist in the different phases? (in minutes)

- Pre-transport
- During transport
- Post-transport

1. Did you check the checklist items by yourself?

- Yes
- No

1. Did you check the checklist items with a second person?

- Yes
- No

1. If the checklist items were checked with a second person, with whom did you check these items?
2. Why do you use the checklist – please specify in your own words?
3. In your opinion, will you recommend the checklist to a colleague?
4. If ‘Yes’ or ‘No’ for question 15, please specify…………………………..
5. Any closing remarks?
